# Alu Methylation Patterns in Type 1 Diabetes: A Case-Control Study

**DOI:** 10.3390/genes14122149

**Published:** 2023-11-28

**Authors:** Andromachi Katsanou, Charilaos A. Kostoulas, Evangelos Liberopoulos, Agathocles Tsatsoulis, Ioannis Georgiou, Stelios Tigas

**Affiliations:** 1 Department of Endocrinology, University of Ioannina, 45110 Ioannina, Greece; katsanoumachi@yahoo.gr (A.K.); atsatsou@uoi.gr (A.T.); 2 Department of Internal Medicine, Hatzikosta General Hospital, 45445 Ioannina, Greece; 3 Laboratory of Medical Genetics, Faculty of Medicine, School of Health Sciences, University of Ioannina, 45110 Ioannina, Greece; chkostoulas@gmail.com (C.A.K.); igeorgio@uoi.gr (I.A.G.); 4 First Department of Propaedeutic Internal Medicine, Medical School, National and Kapodistrian University of Athens, Laiko General Hospital, 11527 Athens, Greece; elibero@med.uoa.gr

**Keywords:** DNA methylation, Alu retroelement, Type 1 diabetes

## Abstract

Evidence suggests that genome-wide hypomethylation may promote genomic instability and cellular senescence, leading to chronic complications in people with diabetes mellitus. Limited data are however available on the Alu methylation status in patients with type 1 diabetes (T1D). **Methods**: We investigated DNA methylation levels and patterns of Alu methylation in the peripheral blood of 36 patients with T1D and 29 healthy controls, matched for age and sex, by using the COmbined Bisulfite Restriction Analysis method (COBRA). **Results**: Total Alu methylation rate (mC) was similar between patients with T1D and controls (67.3% (64.4–70.9%) vs. 68.0% (62.0–71.1%), *p* = 0.874). However, patients with T1D had significantly higher levels of the partial Alu methylation pattern (mCuC + uCmC) (41.9% (35.8–45.8%) vs. 36.0% (31.7–40.55%), *p* = 0.004) compared to healthy controls. In addition, a positive correlation between levels of glycated hemoglobin (HbA1c) and the partially methylated loci (mCuC + uCmC) was observed (Spearman’s rho = 0.293, *p* = 0.018). Furthermore, significant differences were observed between patients with T1D diagnosed before and after the age of 15 years regarding the total methylation mC, the methylated pattern mCmC and the unmethylated pattern uCuC (*p* = 0.040, *p* = 0.044 and *p* = 0.040, respectively). **Conclusions**: In conclusion, total Alu methylation rates were similar, but the partial Alu methylation pattern (mCuC + uCmC) was significantly higher in patients with T1D compared to healthy controls. Furthermore, this pattern was associated positively with the levels of HbA1c and negatively with the age at diagnosis.

## 1. Introduction

Type 1 diabetes (T1D) is an autoimmune disease, characterized by the presence of islet autoantibodies, insulitis and progressive islet β-cell destruction leading to hyperglycemia and eventually chronic vascular complications. Genetic susceptibility is a significant factor in the pathogenesis of the disease, and several T1D loci have been identified so far, most notably the MHC locus [1,2,3]. However, factors other than genetics seem to contribute to the development of the disease, as suggested by the fact that the incidence of T1D has been doubling every twenty years for the past few decades. This trend cannot be solely attributed to genetic factors, indicating a powerful impact of environmental and epigenetic factors [4].

The major mechanisms of epigenetic genome modifications are DNA methylation, histone modification and non-coding RNA action. The role of epigenetic mechanisms in the pathogenesis of T1D has been extensively investigated; DNA methylation and microRNA have been suggested as biomarkers to predict islet β-cell death. Current evidence suggests that alterations in DNA methylation status may contribute to the increasing prevalence of T1D [5,6,7,8,9,10].

In addition to gene polymorphisms, changes in the structure of the genome, which could be due to repeated elements or endogenous viral elements (EVEs), may play a role in the susceptibility to T1D. These elements encompass human endogenous retroviruses (HERVs) and non-long terminal repeat (non-LTR) retrotransposons, which include long and short interspersed nuclear elements [11,12,13,14]. Most high copies of non-LTR retroelements are long interspersed nuclear elements (LINEs) and short interspersed nuclear elements (SINEs) such as the Alus, constituting 16.9% and 10.6% of the human genome, respectively [15]. Certain Alu elements move to different genomic locations by means of the protein encoded by the second open reading frame (ORF2) of LINE-1 [16]. Alu elements contain their own transcriptional signals and are frequently incorporated into the transcriptome via exonization [17]. Previous research suggested that hypomethylation of Alu and LINE-1 are the reasons behind global hypomethylation and genomic instability in several types of cancer and autoimmune diseases [18,19,20]. As a result of the increasing use of whole-genome sequencing for diagnostic purposes, a possible role of retrotransposons in the pathogenesis of several conditions has emerged [21]. Specifically, hypomethylation of Alu, LINE-1 and HERV have been observed in cancer, embryogenesis [22], aging [23], congenital malformation [24], exposure to certain environmental factors [25], smoking [26], nutritional deficiencies [27] and autoimmune diseases [28,29,30].

Although Alu hypomethylation appears to serve as a cellular senescence biomarker [31,32] promoting genomic instability [33,34] and numerous studies suggest that Alu repeat elements are implicated in several diseases, there is sparse information available regarding their potential functional and biological significance in T1D. In a genome-wide sequence analysis, a significant enrichment of Alu retroelements within the T1D candidate genes was observed, proposing that inverted Alus (IRAlus) might be involved in regulating the expression levels of the host genes in patients with type 1 diabetes [35]. According to a recent study, Alu methylation levels could serve as a useful biomarker for monitoring cellular senescence in patients with T2D as Alu hypomethylation was found to be directly correlated with high fasting blood glucose and glycated hemoglobin (HbA1c) [32]. However, limited data are currently available on the Alu methylation status and its potential association with hyperglycemia and HbA1c in patients with T1D.

The aim of this study was to investigate (a) the total methylation and the different methylation patterns of Alu elements in patients with T1D compared to healthy controls and (b) whether glycemic control is associated with the methylation status in patients with T1D.

## 2. Materials and Methods

**Study design and participants**: In the present case-control study, the combined bisulfite restriction analysis (COBRA) method was used to assess the DNA methylation levels and Alu methylation patterns in the peripheral blood in patients including T1D patients and healthy controls, matched for age and sex. We included 36 patients with T1D who consecutively attended the outpatient clinic of the Department of Endocrinology and Diabetes, in the University Hospital of Ioannina, and 29 age and sex-matched normal, healthy subjects with no significant past medical history, who attended the outpatient clinic for a routine health examination. Exclusion criteria were history of cancer or autoimmune disease [28], smoking [26], uncontrolled hypertension (>140/90 mmHg) and obesity or overweight (body mass index > 25 kg/m^2^) [36,37,38,39,40], since the above factors are known to affect the methylation status of retroelements [39,41] or are possibly related to the pathogenesis of T1D [42]. The study was approved by the ethics committee of Ioannina University Hospital (approval number #1586), and all participants provided informed consent prior to study enrollment, in accordance with the Helsinki Declaration.

**Figure 1:**
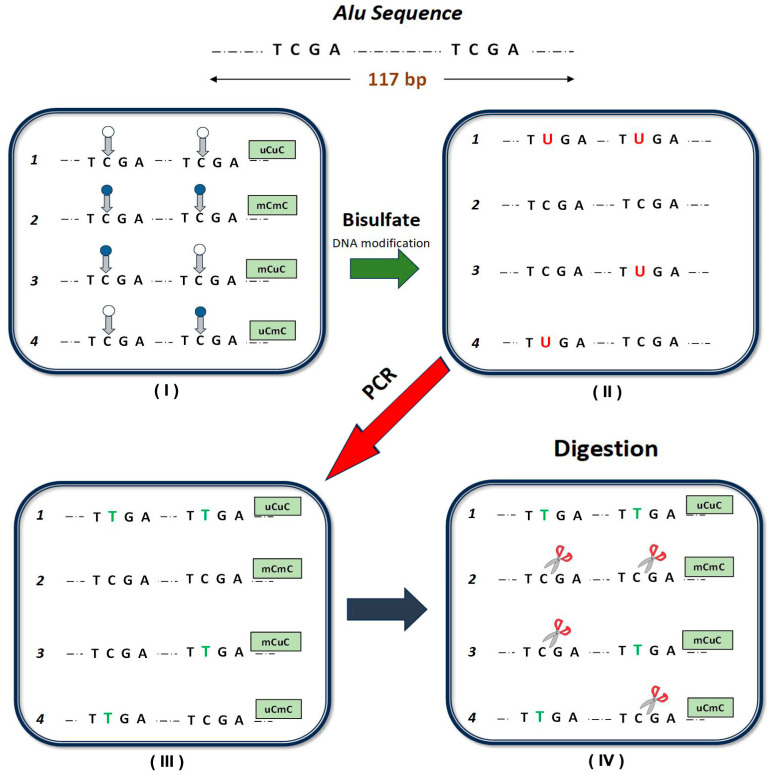
Schematic description of the COBRA-interspersed repetitive sequence PCR. The blue circles represent methylated cytosine and the hollow ones represent unmethylated cytosine (**I**). By bisulfate DNA modification, the unmethylated cytosine is converted to uracil, while methylated cytosine remains cytosine (**II**). This is followed by PCR (**III**) and digestion (**IV**). Scissors represent positions where enzyme Taq1 specifically identified methylated cytosine. The possible methylation patterns for Alu amplicons include hypermethylated (mCmC), hypomethylated (uCuC) and partially methylated loci (mCuC and uCmC). In each model, enzyme Taq1 specifically identified methylated cytosine.

**Laboratory methods**: COBRA-interspersed repetitive sequence PCR is a highly accurate semi- quantitative methylation measurement, able to detect more than one CpG site, even when pyrosequencing cannot demonstrate a DNA methylation pattern [15,43]. **DNA extraction and bisulfate DNA modification**: DNA was extracted from peripheral blood cells using the DNA Blood Mini kit (Qiagen, Hilden, Germany) according to the manufacturer’s protocol. DNA concentration was measured using a Quawell Q500 spectrophotometer. A bisulfite EpiTech methylation kit (Qiagen, Germany) was used for the conversion of 1 mg of the extracted DNA according to the manufacturer’s protocol. To assess methylation levels of Alu, the sodium-bisulfite-treated DNA in each sample was amplified by PCR using 0.2 mM of deoxynucleotide triphosphate, 1 mM of magnesium chloride, 1 U of HotStarTaq DNA Polymerase (Qiagen, Germany) and 0.3 μM primer pairs (Alu-F: 5′-GGCGCGGTGGTTTACGTTTGTAA-3 and Alu-R: 5′-TTAATAAAAACGAAATTTCACCATATTAACCAAAC-3′) (Figure 1).

**Figure 2:**
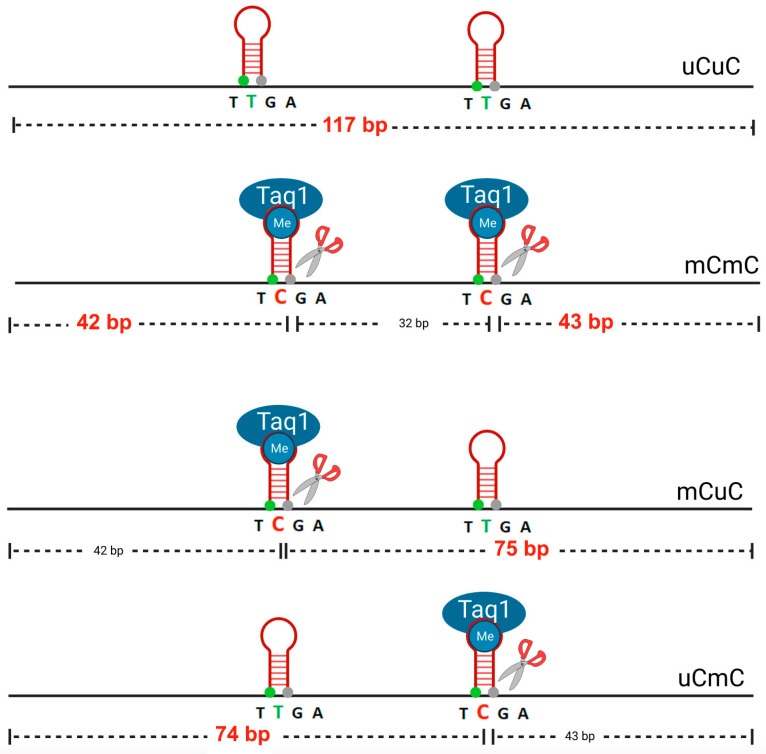
Digestion: In each model, enzyme Taq1 specifically identified methylated cytosine. The different methylation patterns of the Alu element resulted in differently sized digested products of 117 bp, 74/75 bp and 42/43 bp, resulting in five bands with distinct sizes of 117, 75, 74, 43 and 42 bp (in red). Scissors represent positions where enzyme Taq1 specifically identified methylated cytosine. The red shapes represent the first position of methylated or unmethylated cytosine.

Analysis with Bisearch showed that the primers will amplify about 3500 PCR fragments, with representative Alus that are scattered throughout the genome. For Alu amplification, we used the following PCR conditions: 95 °C for 15 min, 40 cycles of 95 °C for 45 s, 57 °C for 45 s, 72 °C for 45 s and a final extension at 72 °C for 7 min. Then, Alu PCR products were digested using 2 U of TaqI enzyme (New England Biolabs, Ipswich, MA, USA) and incubated at 65 °C overnight (Figure 2). Finally, the digested PCR products were analyzed by 8% acrylamide gel and SYBR stain (Lonza, Biologics Inc. Houston, TX, USA), and the intensity of the Alu methylation band was observed and measured by phosphorimager by using 1D image analysis software (kodaks v3.6.4, KODAK, Rochester, NY, USA).

**Methylation analysis**: The COBRA-Alu PCR product generated a mix of the patterns based on the methylation status of the two CpG dinucleotides studied: (1) an unmethylated pattern uCuC (indicating hypomethylation at both CpGs), (2) two partially methylated patterns (mCuC and uCmC) and (3) a methylated pattern mCmC (indicating hypermethylation at both CpGs).

Further discrimination of the three patterns was achieved by subsequent digestion of the generated mix with Taq1 and resulted in five bands with distinct sizes of 117, 75, 74, 43 and 42 bp. Each pattern is represented by a combination of bands generated by the Taq1 digestion (Figure 2).

The Alu methylation level of each pattern was calculated to obtain the exact percentage. The calculation was performed as follows: Initially, the intensity of each band was divided by the length (bp) of the double-stranded DNA: intensity of the 117 bp band divided by 117 (% intensity 117 bp/117 = A), intensity of the 74 bp and 75 bp band divided by 74.5 (% (intensities 74 bp + 75 bp)/74.5 = B), intensity of the 42 bp and 43 bp band divided by 43.5 (% (intensities 43 bp + 42 bp)/43.5 = D) and D − B = C (C = hypermethylated loci, mCmC). Then, the Alu methylation frequency in each pattern was calculated as follows:

- Total methylated loci % mC = 100 × (2C + 2B)/(2A + 2B + 2C) = 100 × (2D)/(2A + 2D);
- Methylated pattern % mCmC = 100 × C/(A + B + C);
- Unmethylated pattern % uCuC = 100 × A/(A + B + C);
- And partially methylated patterns (% uCmC + % mCuC) =100 × B/(A + B + C).

The same 3 bisulfite-treated 3 Daudi (Leukemia), Jurkat (Leukemia) and HeLa (cervical cancer) DNA were used as positive controls in every experiment to reduce inter-assay variability of COBRA. Laboratory processing and analysis as well as blood sample collection from study participants was performed in the Laboratory of Medical Genetics of Ioannina University.

The total Alu methylation rate (mC) was compared between cases and controls, as well as the prevalence of each of the three Alu different methylation patterns: 1. hypermethylated pattern (mCmC), 2. hypomethylated pattern (uCuC) and 3. partially methylated patterns (mCuC and uCmC). In addition, we performed analysis of total methylation and patterns according to sex and age at diagnosis.

**Statistical analysis**: To test the fitting of continuous variables to normal distribution, the Shapiro–Wilk test was used. Continuous variables with non-normal distribution were presented as medians and corresponding interquartile ranges (IQRs), while all dichotomous variables were presented as percentages. Differences between cases and controls regarding baseline characteristics and Alu methylation patterns were assessed with the use of the nonparametric methods of the Mann–Whitney test and Chi-squared test, as appropriate. The association of Alu methylation and patterns with available patient characteristics was further assessed with the use of Spearman’s correlation coefficient. Also, we used the Spearman’s correlation coefficient to evaluate the correlation among all possible methylation patterns. All statistical analyses were performed with the SPSS version 25 by DatAnalysis. A *p*-value of 0.05 was set as the threshold for statistical significance.

Finally, we categorized the patients in two groups according to the age at diagnosis of T1D (0–15 years and ≥15 years) to assess the possible epigenetic role of Alu retroelement in childhood and in later-onset T1D, as younger age at diagnosis may reflect a greater influence of genetic factors in the disease process [1,38,42,44].

## 3. Results

The characteristics and ALU methylation patterns of study participants are shown in Table 1. As expected, patients with T1D had significantly higher fasting glucose (116.5 (95.5–170.5) vs. 82.0 (76–90), *p* < 0.001) and HbA1c levels (7.5% (6.8–8) vs. 5.1% (4.8–5.3), *p* < 0.001) compared to controls. The median age at diagnosis of T1D was 14 years (9.5–21.0), corresponding to a median of 12 (4.0–20.5) years of disease duration at the time of enrollment into the study.

### 3.1. Investigation of the Total Methylation and the Different Methylation Patterns of Alu Elements in Patients with T1D Compared to Healthy Controls

**Figure 3:**
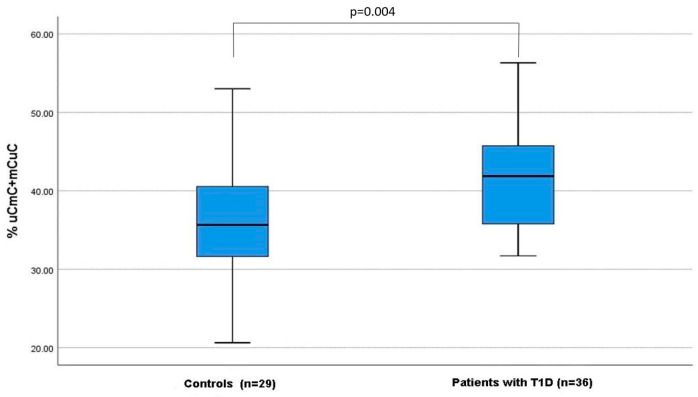
Percentage of partially methylated Alu pattern (mCuC + uCmC) in patients with T1D and healthy controls. Results are presented as box plots: the boxes represent the interquartile ranges (25th to 75th percentile), the median lines represent the 50th percentile and the whiskers represent the minimum and maximum values.

No significant differences between patients with T1D and age, sex-matched controls were identified in the total mC (67.3% (64.4–70.9%) vs. 68.0% (62.0–71.1%), *p* = 0.874). However, the partial Alu methylation (mCuC + uCmC) pattern was more frequently observed in T1D patients compared to controls (41.9% (35.8–45.8%) vs. 36.0% (31.7–40.55%), *p* = 0.004) (Figure 3). The prevalence of hypermethylated (mCmC) and hypomethylated (uCuC) Alu methylation patterns was similar in T1D patients and controls (Table 1).

### 3.2. Investigation of the Association of Alu Methylation and Patterns with Patients’ Characteristics and Glycemic Control (Table 2)

**Figure 4:**
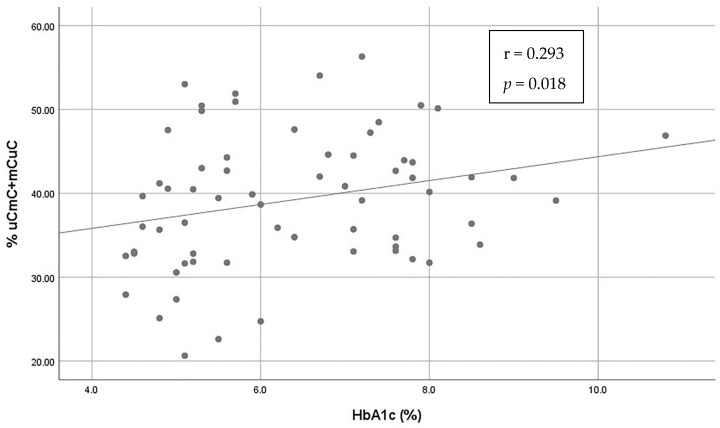
Scatter plot and Spearman correlation of the partially methylated Alu pattern (mCuC + uCmC) with HbA1c levels in patients with T1D. Correlation coefficients (r) and *p* values are depicted.

In correlation analyses, a positive correlation between HbA1c and the partially methylated loci (mCuC + uCmC) Alu pattern was observed (Spearman’s rho = 0.293, *p* = 0.018) (Figure 4). No other association of the different Alu methylation patterns with age, sex, HbA1c, fasting glucose, duration of diabetes or the presence of chronic diabetes complications was detected (Table 2). As far as age at diagnosis of T1D is concerned, a positive correlation with the mCmC pattern (Spearman’s rho = 0.428, *p* = 0.009) and a negative correlation with the partially methylated (mCuC + uCmC) pattern (Spearman’s rho = −0.431, *p* = −0.009) were observed. Statistically significant results are presented in bold (Table 2).

**Table 2:** Correlation analyses on the association of different Alu methylation patterns with patient characteristics.

Variable		mC (%)	mCmC (%)	uCmC + mCuC (%)	uCuC (%)
**Age**		rho = 0.214, *p* = 0.087	rho = 0.149, *p* = 0.235	rho = 0.09, *p* = 0.474	rho = −0.213, *p* = 0.089
**Sex**	**Female**	66.9 (62.6–70.1)	25.57 (19.4–36.3)	41.2 (33.5–44.5)	33.0 (30–37.5)
	**Male**	68.0 (64.3–71.9)	29.3 (20.0–35.6)	39.4 (33.0–42.7)	32.0 (28.0–36.0)
		MW = 500, *p* = 0.812	MW = 480, *p* = 0.615	MW = 450, *p* = 0.368	MW = 501, *p* = 0.821
**Age of diagnosis**		rho = 0.250, *p* = 0.142	rho = 0.428 **, *p* = 0.009	rho = −0.431 **, *p* = −0.009	rho = −0.256, *p* = 0.131
**Duration of disease**		rho = 0.134 *p* = 0.437	rho = 0.061 *p* = 0.723	rho = 0.088 *p* = 0.611	rho = −0.128 *p* = 0.456
**Presence of chronic** **diabetes complications**	**No**	66.3 (62.1–69.8)	25.6 (17.4–34.7)	40.9 (33.9–46.9)	33.5 (30.0–38.0)
	**Yes**	68.5 (65.3–71.4)	24.0 (20.0–27.4)	42.3 (40.2–44.5)	31.5 (29.0–35.0)
		MW = 117, *p* = 0.240	MW = 148, *p* = 0.860	MW = 119, *p* = 0.267	MW = 116, *p* = 0.240
**Fasting glucose**		rho = −0.110, *p* = 0.381	rho = −0.178, *p* = 0.156	rho = 0.187, *p* = 0.136	rho = 0.117, *p* = 0.355
**HBA1c**		rho = 0.088, *p* = 0.486	rho = −0.135, *p* = 0.283	rho = 0.293 *, *p* = 0.018	rho = −0.086, *p* = 0.498

Abbreviations: rho = Spearman’s rank correlation coefficient; MW = Mann–Whitney U statistic *. Correlation is significant at the 0.05 level (2-tailed) **. Correlation is significant at the 0.01 level (2-tailed)**.** *p*-values in bold text indicate a statistically significant difference.

In addition, the association of total methylation and the different methylation patterns of Alu elements was assessed between patients and controls. In the group of patients with T1D, we noticed a positive correlation between mC and mCmC (rho = 0.704, *p* < 0.001) and a negative correlation between (uCmC + mCuC) and mC (rho = −0.448, *p* = 0.006). Likewise, in the control group, a positive correlation between mC and mCmC (rho = 0.664, *p* < 0.001) was observed, while (uCmC + mCuC) was not associated with mC (rho = −0.114, *p* = 0.555).

### 3.3. Differences between Patients with T1D Diagnosed before and after the Age of 15 Years

Using the non-parametric Mann–Whitney test, significant differences were observed between patients with T1D diagnosed before and after the age of 15 years in the total methylation mC, the methylated pattern mCmC and the unmethylated pattern uCuC (*p* = 0.040, *p* = 0.044 and *p* = 0.040, respectively) (Table 3).

## 4. Discussion

This study investigated Alu total methylation (mC) and its different methylation patterns in patients with T1D compared to healthy controls. No significant differences between patients with T1D and age- and sex-matched controls were identified in the total mC, but patients with T1D had significantly higher levels of the partial Alu methylation (mCuC + uCmC) pattern. Furthermore, this pattern was positively associated with the levels of HbA1c and negatively with the age at diagnosis of diabetes. No correlation between mC and glycemic status (fasting glucose or HbA1c) was observed. In addition, no correlation between the duration of disease or complications and the total methylation or methylation patterns was detected. Moreover, a negative association between the pattern (uCmC + mCuC) and mC was observed in patients with T1D, but not in the control group, indicating that this change in methylation status was specific for patients with T1D.

Recent evidence suggests that not only genetic but also environmental influences on T1D genetic loci are mediated by differential levels or patterns of DNA methylation (hypo or hypermethylation) [8,45,46]. For example, some of the genes that have been identified to be associated with T1D, such as *GAD2* and *HLA-DQB1*, were found to be hypomethylated or hypermethylated [7]. Also, a genome-wide analysis of the DNA methylation quantitative trait locus (mQTL) in human pancreatic islets revealed 383 CpGs (0.08% of tested CpGsites), showing significant associations including known diabetes loci, e.g., *ADCY5*, *KCNJ11*, *HLA-DQA1*, *INS*, *PDX1* and *GRB10*. Further functional analyses have indicated that the candidate genes *(GPX7*, *GSTT1* and *SNX19*) have a direct impact on crucial biological functions, such as proliferation and apoptosis of pancreatic β cells [6]. Importantly, individuals who carry the T1D risk variant exhibited less variability in methylation at the CpG residues in intron 1, presumably making them less responsive to cytokines. On the other hand, individuals who carry the protective variant demonstrated greater methylation variability, presumably increasing their sensitivity to cytokines, suggesting that the two populations may respond differently to environmental factors [45].

In previous studies, global DNA methylation was analyzed in MZ twins concordant and discordant for T1D and an overall rise in DNA methylation was observed in affected twins [4].The methylation patterns in the insulin promoter have been examined by Fradin et al. The authors described variations in methylation between patients with T1D and healthy individuals and identified a 3-CpG-hypomethylation pattern that seemed to be present only in these patients. Moreover, CpG methylation appeared to have no correlation with HbA1c or T1D duration [46].

In a recent study aiming to outline differences in methylation patterns between T1D and healthy controls, the regions or sites or that were differentially methylated between the samples from patients with T1D and healthy controls encompassed 84 genes. Among these, 18 genes were already known to exhibit differential methylation in T1D [47]. On the other hand, in another study, no differences in the umbilical cord blood methylation patterns were observed between children who progressed to T1D and those who remained healthy [48].

According to our findings, Alu partial methylation pattern (mCuC + uCmC) was positively associated with the levels of HbA1c. Recent studies have indicated that DNA methylation of retroelements is linked with an increased risk of deteriorating carbohydrate metabolism, obesity and cardiometabolic diseases [36,37,38,39]. In patients with T2D, Alu hypomethylation was found to be directly correlated with high fasting blood glucose and HbA1c. To the best of our knowledge, evidence about any possible correlation between Alu methylation and HbA1c in patients with T1D is missing.

Indeed, DNA methylation has been cross-sectionally associated with T2D and HbA1c in the general population, but longitudinal data and data on T1D diabetes are currently very limited. A study in umbilical vein endothelial cells (HUVEC) revealed that endogenous Alu RNA accumulation during hyperglycemia induced oxidative stress and malfunction in the endothelial cells by inhibiting the expression of superoxide dismutase 2 (SOD2) and endothelial nitric oxide synthase (eNOS) at both the transcription and translation levels through the NFκB signaling pathway. This data indicated that endogenous dsRNA homologous to the Alu Sc subfamily accumulated in hyperglycemic HUVEC endothelial cells [49].

Recently, as far as the role of the DNA methylation in the pathogenesis of T1D is concerned, there has been a growing interest in measuring the levels of unmethylated preproinsulin DNA as an indicator of β cell death. β cells are characterized by a higher number of unmethylated CpG sites within the preproinsulin DNA sequence compared to other cells. It has been observed that patients with newly diagnosed T1D have elevated levels of unmethylated preproinsulin DNA in their peripheral blood samples when compared to healthy individuals [9].

Of note, in a recent epigenome-wide association study (EWAS) in a T1D cohort, loci with DNA methylation (DNAm) were found to be associated with HbA1c. Specifically, DNAm at cg19693031 (Chr 1, *Thioredoxin-Interacting Protein* (*TXNIP*)) and cg21534330 (Chr 17, *Casein Kinase 1 Isoform Delta*) was found to have an inverse relationship with concurrent HbA1c, suggesting that lower methylation levels of *TXNIP* are linked to poorer glycemic control in T1D [50].

In the Diabetes Control and Complications Trial and in the Epidemiology of Diabetes Interventions and Complications follow-up study, DNAm at key CpG sites seemed to play a mediating role in the association between hyperglycemia and complications in metabolic memory by altering the enhancer activity at myeloid and other cells [51].

A zebrafish study suggested that DNA hypomethylation due to hyperglycaemia was heritable [52]. A study of DNA methylation in pancreatic duodenal homeobox 1 (PDX-1), a transcription factor that plays a role in pancreas development and function, reported that hyperglycaemia increased methylation and decreased gene expression of PDX-1 in islets of patients with diabetes [53].

According to a study in patients with T2D, Alu methylation was lower compared to the general population, but no significant difference was found between patients with prediabetes and controls [32]. Also, Alu methylation in patients with T2D progressively decreased with increasing HbA1c levels during a 4-year follow-up period. The authors proposed that this observation suggested a potential link between Alu hypomethylation and the underlying molecular processes, as prolonged hyperglycemia may cause an imbalance in oxidative production and suppression, also induces p53 and may contribute to impaired insulin signaling [43].

In our study, a negative correlation with the partially methylated (mCuC + uCmC) pattern but no correlation with mC and the age at diagnosis of T1D was detected. Levels of mC were higher in patients with T1D over 15 years old. Limited information is available about the retroelements’ role in TD1 pathology during aging. The identification of methylation differences that occur before the onset of islet autoimmunity and clinical diagnosis could imply a role for epigenetics in T1D pathogenesis. For example, this is supported by a study that analyzed multiple blood samples taken before the disease onset from 87 T1D patients and 87 control subjects, revealing that changes in methylation with age were different between cases and controls in ten regions. Moreover, some methylation differences were detectable from birth, either before or after the onset of preclinical islet autoimmunity [54]. In another study, in individuals with T1D-specific autoantibodies, several distinct regions of altered methylation were discovered in CD8+T cells, CD4+T cells and CD4-CD8- cell fractions when compared to control participants. The examination of samples taken before the appearance of antibodies revealed DNA methylation patterns at the very early stage of the disease, including changes in methylation at the promoter of IRF5 in CD4+T cells [55]. During aging, global hypomethylation events are enriched for repetitive sequences and thought to be responsible for the reactivation of retrotransposon elements, so that epigenomic alterations are found in age-associated noncommunicable diseases, such as diabetes mellitus and osteoporosis [56,57]. Consequently, it seems that a later onset of T1D may result in a larger difference in methylation status compared to an earlier disease onset, when the exposure to specific environmental factors that trigger T1D pathogenesis may play a more crucial role than genetic ones. Specifically, the earlier onset of T1D seems to be related with lower mC compared to a later onset. Moreover, the hypothesis that environmental factors play a significant role in the onset of the disease is also supported by the seasonal variation of incidence of T1D. Diagnosis during childhood implies a more aggressive role for genetics, as adult-onset diabetes progresses at a slower rate [58,59].

Finally, our findings are consistent with previous work reporting that sex does not influence Alu methylation. For example, a recent study investigating the Alu methylation levels of white blood cells of patients with T2D, pre-T2D and controls has shown that Alu methylation levels were not significantly different between males and females [32]. Also, LIMMA analysis which was performed by comparing the DNA methylation profiles of males vs. female twins did not show any significant alterations of methylation related to sex [5].

To the best of our knowledge, the present study is the first to investigate Alu element methylation and patterns in patients with T1D compared to healthy controls. The method used was COBRA-interspersed repetitive sequence PCR, which is a highly accurate semi- quantitative methylation analysis, able to detect more than one CpG site. However, several limitations should be taken into consideration. Firstly, due to the limited sample size, differences in other methylation patterns between patients with T1D and controls and potential associations of methylation patterns with patient characteristics could have been under-detected. Second, in our analyses we could not account for environmental factors, such as physical activity, diet and exercise, which are known to alter the human epigenome. Third, we did not collect data on other medical comorbidities in T1D patients or concurrent medication, and thus the potential role of these factors in Alu methylation cannot be estimated or controlled. Finally, the use of a cutoff age of 15 years to explore potential differences in Alu methylation and methylation patterns between patients with disease onset at a young versus older age is partly arbitrary.

## 5. Conclusions

Our results suggest that total Alu methylation rates were similar, but the partial Alu methylation pattern mCuC + uCmC was significantly higher in patients with T1D compared to healthy controls. Furthermore, this pattern was positively associated with the levels of HbA1c and negatively with the age at diagnosis. Patterns of DNA methylation may serve as useful markers of epigenetic changes in addition to DNA methylation levels in T1D. Our findings need to be further tested in large-scale, observational studies on one or more retroelements.

## Figures and Tables

**Table 1:** Characteristics and Alu methylation patterns in patients with T1D and controls.

Variable	Patients with T1D	Controls	*p*-Value
	(*n* = 36)	(*n* = 29)	
**Subject Characteristics**			
Age (years, median, IQR)	27.5 (19.0–38.0)	29.0 (22.0–33.0)	0.905
Males/Females (%)	55.60/44.4	58.60/41.4	0.804
Systolic blood pressure (mmHg, median, IQR)	120.0 (110.0–130.0)	115.0 (100.0–125.0)	0.325
Diastolic blood pressure (mmHg, median, IQR)	75.0 (70.0–80.0)	75.0 (70.0–80.0)	0.761
BMI (kg/m^2^)	22.0 (21.0–23.0)	22.2 (21.0–23.0)	0.599
Fasting glucose (mg/dl, median, IQR)	116.5 (95.5–170.5)	82.0 (76.0–90.0)	<0.001 *
HbA1c (%,median, IQR)	7.5 (6.8–8.0)	5.1 (4.8–5.3)	<0.001 *
**Methylation Patterns**			
mC (%, median, IQR)	67.3 (64.4–70.9)	68.0 (62.0–71.1)	0.874
mCmC (%, median, IQR)	24.8 (18.1–34.0)	32.9 (23.3–37.9)	0.073
uCmC + mCuC (%, median, IQR)	41.9 (35.8–45.8)	36.0 (31.7–40.55)	0.004 *
uCuC (%, median, IQR)	33.5 (29.5–36.0)	32.0 (29.0–38.0)	0.916

Abbreviations: T1D = type 1 diabetes mellitus; IQR = interquartile range. *. Correlation is significant at the 0.05 level (2-tailed). *p*-values in bold text indicate a statistically significant difference.

**Table 3:** Correlation analyses between the methylation patterns according to the age of diagnosis of T1DM (Mann–Whitney test).

Methylation	Age at Diagnosis	*n*	Median (25th–75th)	U	*p*-Value
**mC**	<15	21	66.64 (63.04–68.72)	86	0.040 *
	≥15	14	70.10 (65.30–72.08)		
**mCmC**	<15	21	20.08 (17.33–27.38)	87	0.044 *
	≥15	14	29.58 (21.37–36.71)		
**uCmC + mCuC**	<15	21	42.70 (39.16–47.62)	97	0.096
	≥15	14	40.01 (34.71–43.93)		
**uCuC**	<15	21	33.00 (31.00–37.00)	86	0.040 *
	≥15	14	30.00 (28.00–35.00)		

*. Correlation is significant at the 0.05 level. *p*-values in bold text indicate a statistically significant difference.

## Data Availability

Data presented in this study are available upon reasonable request from the corresponding author. The data are not publicly available due to ethical reasons.
